# Genome-Wide Identification and Analysis of *BrTCP* Transcription Factor Family Genes Involved in Cold Stress Tolerance in Winter Rapeseed (*Brassica rapa* L.)

**DOI:** 10.3390/ijms252413592

**Published:** 2024-12-19

**Authors:** Yanxia Xu, Li Ma, Xiucun Zeng, Yaozhao Xu, Xiaolei Tao, Abbas Muhammad Fahim, Lijun Liu, Junyan Wu, Gang Yang, Yuanyuan Pu, Tingting Fan, Wangtian Wang, Wancang Sun

**Affiliations:** 1 State Key Laboratory of Aridland Crop Science, College of Agronomy, Gansu Agricultural University, Lanzhou 730070, China; xyx7991@163.com (Y.X.); txl162185@163.com (X.T.); fahimabbaskhan@yahoo.com (A.M.F.); liulj198910@163.com (L.L.); wujuny@gsau.edu.cn (J.W.); yangang1018@163.com (G.Y.); vampirepyy@126.com (Y.P.); fantt@gsau.edu.cn (T.F.); wangw@gsau.edu.cn (W.W.); sunwanc@gsau.edu.cn (W.S.); 2 College of Life Sciences and Engineering, Hexi University, Zhangye 734000, China; xuyaozhao@126.com

**Keywords:** *Brassica rapa*, TCP transcription factor, abiotic stress

## Abstract

TCP transcription factors are important during plant growth and stress responses. However, their role in the cold stress response of *Brassica rapa* L. remains poorly understood. In this research, we identified the *TCPs* gene family in *B. rapa* to learn the features of the *BrTCP* gene family, functionally annotating the interacting proteins of TCP4 and analyzing their expression levels. Our results illustrated the presence of 19 members of the *BrTCPs* family in *B. rapa*, exhibiting molecular weights ranging from 27,367.45 to 59,433.64 Da. All identified proteins were classified as unstable, with isoelectric points ranging from 5.5 to 9.48. Subcellular localization forecasted that TCP proteins were all positioned in the nucleus. The *BrTCP* gene structure is relatively simple, with only seven members possessing introns, and none of the members contain UTR regions. *BrTCPs* comprise hormone-, light-, and stress-responsive elements. We found that the frequency of photoresponsive elements was greatest in the promoter region, suggesting that *BrTCP* genes are regulated by light signals and function synergistically with plant growth and development. In addition, five candidate interaction proteins of *BrTCP4* were identified using yeast two-hybrid screening. RNA-Seq and q-PCR analyses of the interacting genes revealed differential expression of *BrTCP* family genes across various tissues following cold stress. Significant responses were observed under low-temperature stress, drought stress, and rehydration treatment, suggesting that these genes play crucial roles as regulators of the molecular network mechanisms responding to stress. This study enhances our understanding of the *BrTCP* family and provides significant insights into the stress tolerance mechanisms of *B. rapa.*

## 1. Introduction

*B. rapa* is classified within the Brassica genus of the cruciferous family, representing the predominant oil crop in China and the primary winter oil crop in northwest China [1], which plays a crucial role in maintaining a steady supply of edible oil and enhancing economic gains [2]. The winter environmental conditions in the northern part of our country are harsh, and the extreme temperature can be as low as −32 °C [3]. This low-temperature environment has a limiting effect on the growth and development of plants in northwest China, making the enhancement of cold tolerance a critical survival tactic for overwintering plants during the cold winter months [4]. Fang Yan et al. studied the differentially expressed genes (DEGs) for amino acid isoaccumulation in Winter rapeseed under low-temperature stress, whereby cold-tolerant and non-cold-tolerant cabbage-type oilseeds were investigated using RNA-seq, with the aim of discovering new genes related to cold tolerance in *B. rapa* [5], which provided a scientific basis for the genetic improvement of *B. rapa* varieties [4].

Transcription factors (TFs) enhance plant resilience to cold stress by scavenging reactive oxygen species, increasing the quantity of ROS, and elevating the expression levels genetically related to cold stress [6]. They can specifically bind to the promoter region of the target gene to regulate the expression of the target gene [7]. TCP proteins are a class of TF specific to plants. They play a role in the dormancy of buds [8], leaf development [9,10], internode elongation [9], fruit ripening [11,12,13,14], and enhancing stress resistance [15,16,17]. They regulate signal transduction and the synthesis and transport of various plant hormones, facilitating adaptation to external environmental influences on plant growth and development [18]. *OsTCP19* modulates abiotic stress responses through the regulation of ABI4 expression levels, while the overexpression of *OsTCP14* enhances cold tolerance in *Oryza sativa* L. [15,17]. The *GhTCPs* genes in *Gossypium* spp. have demonstrated notable upregulation when exposed to drought, heat, and salt stresses [16]. TCP expression varies across different plant organs, including flowers, leaves, buds, and fruits, as observed in *Vitis vinifera* L. [14] and *Manihot esculenta Crantz* [19]. The amino acid sequence is used as the standard, and the *TCP* gene family is classified into two main groups: Class I (TCP-P/PCF) and Class II (TCP-C). Class II is divided into two subgroups: CIN and CYC/TB1 [20,21]. To date the research on TCP transcription factors mainly focuses on *Arabidopsis thaliana* [22], *Nicotiana tabacum* L. [23,24], *Musa acuminata* L. [25], and *Vitis vinifera* L. [14]. In *A. thaliana*, *AtTCP3* and *AtTCP4* regulate the differentiation of various xylem ducts [26], while *AtTCP14* and *AtTCP15* affect internode length and mediate gibberellin-promoted seed germination in *A. thaliana* [9]. Additionally, a study of TCP transcription factors in *Chrysanthemum morifolium Ramat* revealed that *CmTCP13* expression in *A. thaliana* plants augmented their resilience to salt stress. When subjected to salt stress, CmTCP13 transgenic plants demonstrated enhanced seed germination, root elongation, seedling development, and chlorophyll levels, while exhibiting reduced relative conductance compared to their wild-type (WT) counterparts. In response to saline stress, the expressio mode of stress-associated genes was upregulated in CmTCP13 transgenic plants. This Indicates that *CmTCP13* serves as a significant function in salt stress tolerance in plants [27]. Leng [13] et al. identified 18 TCP transcription factors in grapevine distributed on 11 chromosomes. RNA-seq and RT-qPCR analyses of *VvTCP* genes in grapevine showed that the expression of most *VvTCP* genes could be suppressed by drought and flooding stress. *ZmTCP42* in corn (*Zea mays*) modulates certain abscisic acid or water-deficit stress-responsive genes and serves as a crucial positive mediator of drought resistance [28]. *OsTCP19*, discovered in rice (*Oryza sativa*), reacts to water scarcity and salinity stress and functions as a vital junction in cellular signaling [17]. Therefore, the related genes of the *TCPs* family are essential in the response to the effect of abiotic stress.

Therefore, in present study, the members of the *BrTCPs* gene family were identified and analyzed, and the physicochemical properties, structural composition, chromosome localization, etc., were systematically analyzed. Moreover, through transcriptome analysis, the expression of *BrTCPs* gene family members under cryogenic treatment was analyzed. At the same time, genes associated with stress were selected and verified with protein screening and fluorescence quantitative RT-qPCR, which offered a conceptual foundation for subsequent comprehensive investigation of the molecular pathways underlying *BrTCPs* gene resilience to environmental stressors and the identification of valuable genetic resources in *B. rapa*.

## 2. Results

### 2.1. Identification and Protein Traits of TCPs Genes

The *TCPs* gene family of *B. rapa* was identified and analyzed using BLAST, HMM, and CDD methods, and nineteen *TCP* genes were identified. The physicochemical properties of 19 *TCP* genes in *B. rapa* were analyzed. The number of amino acids was 246 (*BrTCP7*) ~538 (*BrTCP8*), the corresponding molecular weight ranged from 27,367.45 to 59,433.64 Da, the instability coefficient was greater than 40, the isoelectric point was ranged from 5.5 to 9.48, and the subcellular localization prediction showed that all members were in the nucleus (Table 1).

### 2.2. Gene Structure Analysis of TCPs Family in B. rapa

**Figure 1:**
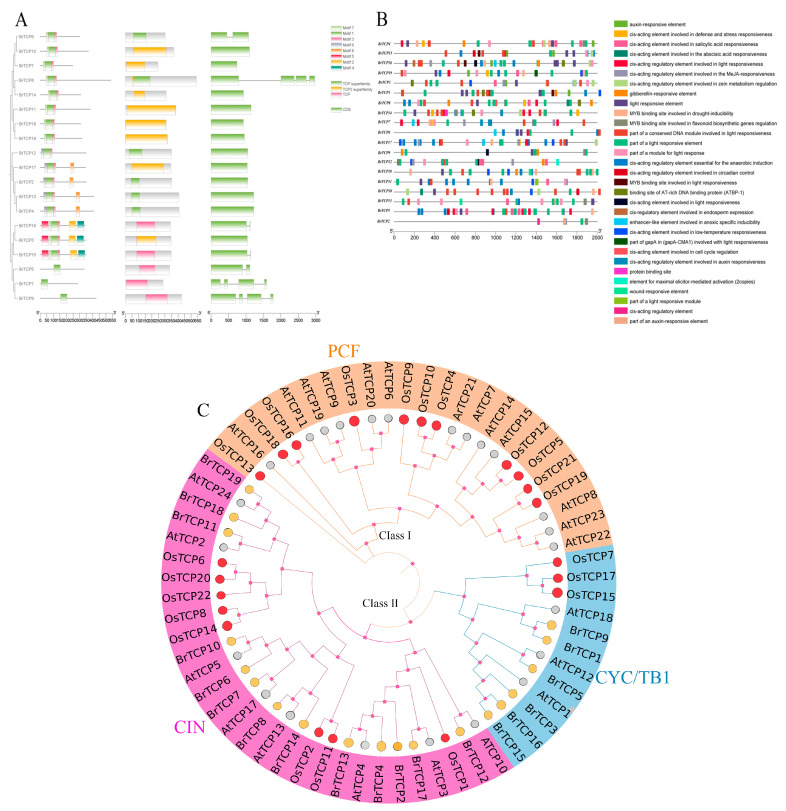
(**A**) Gene structure and conserved protein motif of the TCPs gene family in *B. rapa*; (**B**) distribution of cis-acting elements; (**C**) systematic evolutionary tree.

Domain, gene structure, and motif analyses of the *TCPs* gene family of *B. rapa* were carried out using TBtools V2.110 [29] and online tools (Figure 1A). The domain analysis revealed that all 19 family members contained TCP conserved structural domains, including TCP superfamily or TCP 2 superfamily conserved structural domains, suggesting that all 19 members are capable of TCP structural functions. The gene structure of the *TCPs* family members was easy, and only seven members had introns, among which *BrTCP8*, *BrTCP1,* and *BrTCP9* contained three introns, and no members contained a UTR region. The motif analysis showed that most of the members contained Motif 1 structure, indicating that Motif 1 is an important structure for *TCPs* gene family members to function. Some motifs only appear in a certain branch. For instance, Motif 4 and Motif 5 are only found in the Class II subfamily, and it is hypothesized that they may be structures specific to this subfamily. Among them, *BrTCP5*, *BrTCP1*, and *BrTCP9* have only Motif 1, suggesting that genes with these motifs may perform a certain role.

### 2.3. Analysis of Cis-Acting Elements of TCPs Gene in B. rapa

The cis-acting elements, to some extent, reflect the transcriptional regulation of genes. The elements in the 2000 bp promoter region upstream of the TCPs gene family in *B. rapa* were analyzed using the Plant CARE website (Figure 1B) and mapped using TBtools. A total of 31 response elements were predicted, with a variety of hormone- and stress-responsive elements present, as well as many light-related components. The main elements involved are photoreaction, salicylic acid reaction, abscisic acid reaction, and cis-acting elements involved in coercion. As can be seen from Figure 1B, TCPs gene family members are regulated by a variety of elements. Among them, the photo-responsive elements and cis-acting elements involved in the abscisic acid reaction are the most abundant. The above promoter analysis indicated that the *TCP* gene family is extensively involved in plant responses to environmental stress and hormones, suggesting that these elements are more conserved in the TCPs gene family of Winter rapeseed and may play an important role in the growth process of *B. rapa*.

### 2.4. Phylogenetic Analysis of the Protein Sequence

In the present study, we used MEGA 7.0 [30] to construct a phylogenetic developmental tree to classify the subfamilies of 65 *TCPs* gene family members from three different crops, including 19 from *B. rapa*, 22 from *Oryza sativa* L., and 24 from *A. thaliana* [31] (see Figure 1C). A total of 65 genes from three crops were distinguished into Class I (PCF subfamily) and Class II, where Class II can be further divided into two clades, being the CIN and CYC/TBI subfamilies. In different subfamilies, *TCP* genes have unequal numbers of genes in different branches. The CIN subfamily clustered the largest number of TCP members with relatively close affinities, including 13 BrTCP proteins, 8 AtTCP proteins, and 8 OsTCP proteins. In *A. thaliana* and rice, most of the *B. rapa* TCP members cluster in the CIN subfamily, but not in the PCF subfamily. The TCP members of *A. thaliana* and rice were mostly clustered in the PCF subfamily, including 11 OsTCP proteins and 13 AtTCP proteins. This asymmetric ramification may arise from the evolutionary pressures that species experience, which also establishes a structural foundation for the functional variety within the *BrTCPs* family.

### 2.5. Analysis of Collinearity and Localization of TCPs Genes in B. rapa

**Figure 2:**
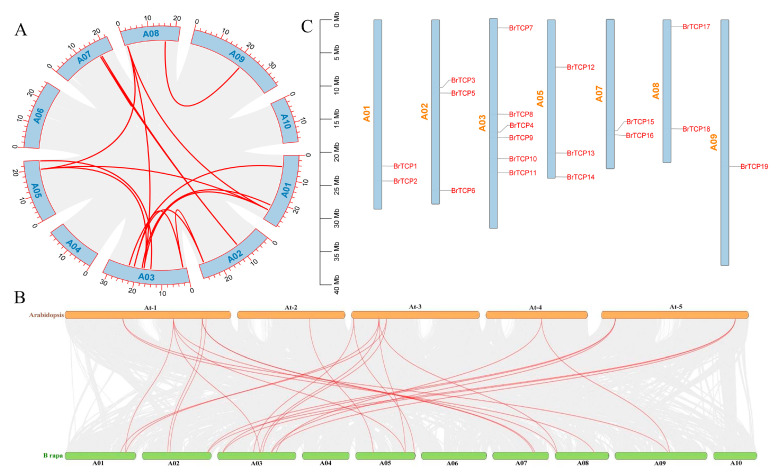
Analysis of collinearity and localization of *TCPs* genes: (**A**) Intraspecies collinearity; (**B**) interspecific collinearity; (**C**) chromosomal localization of Br*TCPs* gene family members.

To gain deeper insight into the development mechanisms of the *TCPs* gene family in B. rapa and analyze the evolutionary relationships among its members, we conducted a collinearity analysis using TBtools software. This investigation aimed to elucidate the evolutionary processes within the *TCPs* gene family in *B. rapa.* The analysis revealed a total of 16 collinear pairs (Figure 2A,B). In the collinearity analysis plot, collinearity of the BrTCP4 gene was observed on all chromosomes except chromosomes A04, A06, and A10. Among them, there were multiple TCPs gene collinearity observations on chromosomes A01, A03, A05, and A08. There is collinearity between four genes and multiple genes. Further analyses of interspecific collinearity using the Arabidopsis thaliana and *B. rapa* genomes revealed phylogenetic relationships among members of the TCP family. There were 27 pairs of TCP collinearity genes composed of 11 AtTCP and 19 BrTCP in the genomes of Arabidopsis thaliana and B. rapa, which were located on chromosomes 1, 2, 3, 5, 7, 8, and 9, respectively.

The 19 of the identified TCPs gene family members were named from BrTCP1 to BrTCP19 based on their location on the chromosome. According to the chromosome distribution results obtained through the visual analysis of TBtools V2.110 (Figure 2C), the TCPs gene family members were mainly distributed on seven chromosomes, among which there were up to six on chromosome A03, followed by A02 (three), A05 (three), A01 (two), A07 (two), and A08 (two), among which the lowest distribution was on chromosome A09, including one gene family member.

### 2.6. Validation of Self-Activation of BrTCP4 and Screening of Interacting Proteins

**Figure 3:**
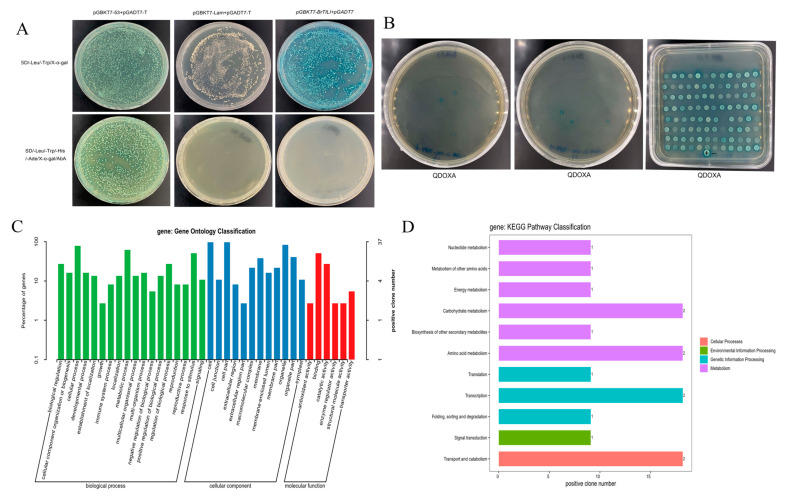
(**A**) pGBKT7-TCP4 yeast self-activation assay; (**B**) screening of pGBKT7-TCP4 interacting proteins; (**C**,**D**) GO annotation and KEGG functional analysis of interacting proteins.

We designed primers based on the CDS sequence of the *TCP4* gene, and the target sequence was ligated into the pGBKT7 vector by enzymatic digestion to construct the pGBKT7-TCP4 bait vector. The bait vector plasmids pGBKT7-TCP4 and pGADT7 were co-transformed into YH2 cold yeast strains. The positive control, pGBKT7-53 + pGADT7-T, exhibited growth and blue coloration on both SD/-Leu/-TrpX-α-gal (DDO/X) and SD/-Leu/-Trp/-His/-Ade/X-α-gal/AbA (QDO/X/A) media (Figure 3A). In contrast, the negative control, pGBKT7-Lam + pGADT7-T, displayed growth on DDO/X medium but failed to grow on QDO/X/A medium. pGBKT7-TCP4 + pGADT7 demonstrated growth and blue coloration on DDO/X medium but did not grow on QDO/X/A medium. This outcome indicates successful transformation of the pGBKT7-TCP4 plasmid into a yeast strain and the absence of self-activation on QDO/X/A, making it suitable for subsequent yeast library screening experiments.

After that, pGBKT7-TCP4 was used as bait, and the yeast cDNA library of European *B. rapa* was screened using the co-transformation method to screen the yeast two-hybrid cDNA library of *B. rapa* and Longyou 7 and screened and cultured on SD/-Leu/-Trp/-His/-Ade/X-alpha-gal/Aba (QDO/X/A) plates, which resulted in the growth and bluing of 96 positive clones (Figure 3B). Yeast vectors were purified from the blue positive transformants cultured on QDO/X/A plates, transferred into *E. coli* for propagation of the positive clone prey vector, and sequenced to determine the sequence information of the positive clones. GO annotation and KEGG functional analysis of the screened interacting proteins revealed (Figure 3C,D) that most of the interacting proteins were involved in cellular components, molecular functions, and biological processes, and their functions were mainly related to carbohydrate metabolism, amino acid metabolism, cellular transport and catabolism, signal transduction, processing of genetic information, and metabolism (amino acid metabolism, biosynthesis, etc.).

### 2.7. Y2H One-to-One Verification of Protein Interactions

**Figure 4:**
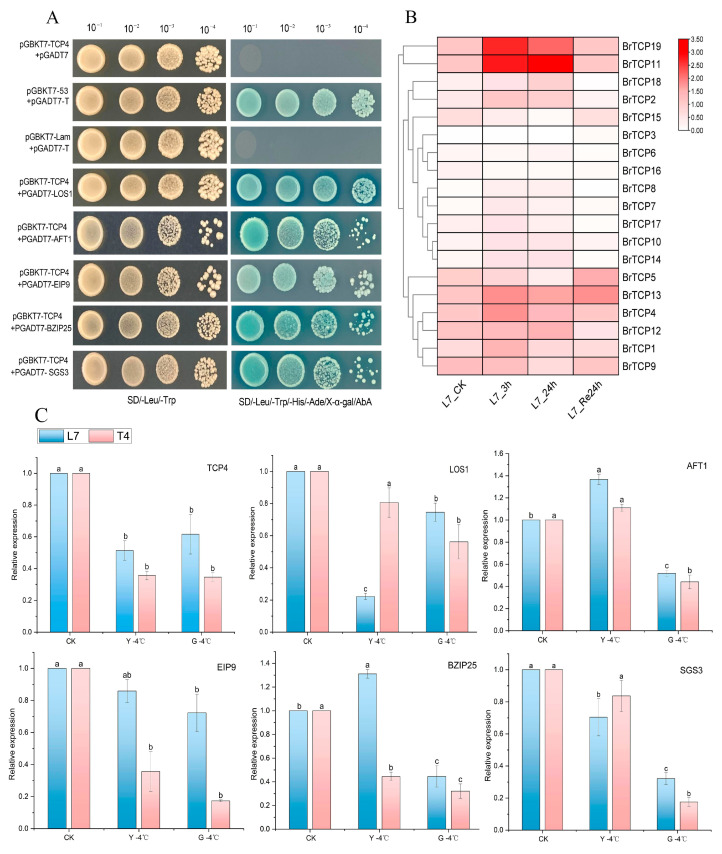
(**A**) One-to-one validation of yeast two-hybrid crosses of BrTCP4 and its reciprocal proteins; (**B**) transcriptome expression after cryogenic treatment; (**C**) expression of BrTCP4 interacting proteins at low temperature. Different lowercase letters indicated significant differences among different treatments (*p* < 0.05).

To further verify that the positive clone was correct, according to the annotation results of the screened positive clones, the expression patterns of abiotic stress, and the literature reports, five candidate interaction proteins were selected for TCP4, the full-length CDS was cloned, the vector pGADT7 was constructed, and the interaction between the five candidate interaction proteins and TCP4 was verified by co-transfecting yeast strain Y2H Gold with the pGBKT7-TCP4 plasmid, respectively. One-to-one validation using yeast showed that the experimental groups pGBKT7-TCP4 + pGADT7-LOS1, pGBKT7-TCP4 + pGADT7-AFT1, pGBKT7-TCP4 + pGADT7-EIP9, pGBKT7-TCP4 + pGADT7-BZIP25, and pGBKT7-TCP4 + pGADT7-SGS3, as well as the positive control and the negative control, could grow bacteria normally on the second-deficient plate DDO, indicating that the plasmid transformation was normal. Except for the negative control, all other co-transformed plasmids could grow and turn blue on QDO/X/A; however, the lower the concentration, the lower the bacterial growth, suggesting that TCP4 interacts with all five candidate interacting proteins in yeast cells (Figure 4A).

### 2.8. Tissue Expression Analysis of BrTCP4 Interacting Proteins at Low Temperatures

The relative expression of BrTCP4 interactions protein genes was analyzed in different parts of Longyou 7 and Tianyou 4 under low-temperature treatment at −4 °C for 8 h (Figure 4B). The outcome revealed that there were differences in the expression of interacting proteins in different parts of plants after cold stress. The manifestation of EIP9 in the root system of Longyou 7 exceeded that of Tianyou 4 when subjected to −4 °C for 8 h, with Longyou 7 exhibiting an EIP9 expression level in its roots that was roughly quadruple that of Tianyou. Among them, the expression of *LOS1* was 4 times higher in the leaves of Tianyou 4 than that of Longyou 7, and the expression of *SGS3* was about 0.14 times higher in the leaves of Tianyou 4 than that of Longyou 7. The expression of *AFT1* was about 0.37 times higher than that of CK in the leaves of Longyou 7, and 0.11 times higher than that of CK in the leaves of Tianyou 4. The expression level of *BZIP25* in Longyou 7 leaf tissue was 2.95-fold higher than that in Tianyou 4. These findings indicate that *BZIP25* is highly expressed in the foliar tissues.

### 2.9. Expression Analysis of the TCPs Gene Family Under Low-Temperature Stress

We analyzed the expression patterns of *BrTCPs* family members in *B. rapa* Longyou 7 under 4 °C cryogenic stress using pre-laboratory transcriptome data (Figure 4B, Supplementary Table S3). The outcome revealed that the expression of each family member varied at different lengths of cold treatment. *BrTCP19* and *BrTCP11* were significantly increased after cold treatment at 4 °C, *BrTCP19* increased by about 4.5 and 2.9 times at 3 h and 24 h, respectivley, and *BrTCP11* increased by about 5 and 6 times after cold treatment at 3 h and 24 h, respectivley. With the prolongation of cold stress, the expression levels of *BrTCP13*, *BrTCP19*, *BrTCP4*, *BrTCP2*, and *BrTCP1* increased first and then decreased. Relative to 24 h of cold treatment, the expression of *BrTCP13*, *BrTCP9*, and *BrTCP5* increased after 24 h of recovery at 22 °C. The rest of the genes showed different degrees of change after different treatment times at 4 °C. However, the magnitude of this change varied with treatment time. Fluctuations in the expression levels of these genes under diverse cold conditions indicate the *BrTCP* family’s participation in orchestrating cold tolerance responses in *B. rapa.* In summary, its *TCPs* family responded significantly under cold stress, indicating that it is an important regulator in the molecular network mechanism in response to adversity stress.

## 3. Discussion

TCP transcription factors are unique to plants and play significant roles in controlling plant growth and morphogenesis [32,33]. Numerous studies have shown that *TCPs* are extensively involved in various aspects of plant development, such as seed sprouting [34,35], flower and leaf development [9,36], and lateral branch growth [29]. The *TCP* gene family has been characterized in numerous plant species, including *A. thaliana* [9,22,37], banana (*Musa acuminata* L.) [25], *Cymbidium* ssp [38], *Solanum melongena* L. [33], *Cucumis sativus* L. [39], rice [40], and many others. However, the distribution of this gene family in *B. rapa* and its biological functions have not been reported so far. In this research, the *TCPs* gene family was characterized and the *BrTCP4* gene was functionally analyzed using various methods in *B. rapa*. The study of multiple plant gene families has significantly expanded due to advances in whole genome sequencing. A total of 19 *BrTCPs* genes were characterized in this study, and phylogenetic analysis revealed that the TCP proteins of *B. rapa* were divided into two classes, with two clades and three branches, being Class I (PCF subfamily) and Class II (CIN and CYC/TBI subfamily). The same was observed when studying the subfamily classification of orchids, sunflowers, dendrobiums, and others [38,41,42]. Most of these *B. rapa* cluster in the CIN subfamily. The analysis of the physicochemical properties of the BrTCP proteins revealed that all the proteins of this family have good hydrophilicity, and there are significant differences in the molecular mass and isoelectric value of the proteins (Table 1). A similar study of the *TCPs* family in *Dendrobium Sw* has been reported [42]. It is different from *B. rapa* as, in Dendrobium orchid studies, most DcaTCP proteins are hydrophobic. Moreover, it is speculated that the variability in the physicochemical properties of TCP proteins may be related to their involvement in the regulation of the development of different tissues and organs of the plant and hormone signaling [43]. The subcellular location of all TCP proteins in *B. rapa* is positioned in the nucleus, which is identical to the distribution of TCP proteins in plants such as tobacco [23] and Andrographis paniculata [44]. It is presumed that the *BrTCPs* gene family of *B. rapa* mainly plays a regulatory role of transcription factors in the nucleus [44].

With the rapid development of bioinformatics and genomics, more and more studies have been carried out on intron-free genes [45,46]. At this stage, there is growing evidence that most eukaryotes have lost introns during evolution. For example, in plants such as *Avena sativa* L. and tea tree, the loss of introns occurred during evolution [47]. The analysis of the gene structure of *BrTCPs* in *B. rapa* showed that only seven members of the *BrTCPs* gene had introns, and no members contained a UTR region. Although providing no benefit in recombination or species evolution, intron-less genes are usually responsive to stress [48]. Intron-less genes are another type of gene, as opposed to genes with interrupted gene sequences, which are not separated by introns and are able to code for proteins continuously. Intron-less genes have been studied, showing that Arabidopsis thaliana *At4g13650* and *At4g14050* regulate RNA editing in mitochondria [49]; and *AtSAUR19* [50] and *AtSAUR63* [51] positively regulate cell expansion by regulating growth hormone transport.

Forecasting promoter elements at the transcriptional level offers a more comprehensive understanding of gene regulation [52]. Examinations of the functional structural domains and promoter cis-acting elements of *BrTCP* indicated that they are equally implicated in these stress response processes. Nevertheless, additional investigations are required to confirm this [53]. The cis-acting elements of 19 *BrTCP* genes were evaluated and discovered to encompass photoreactive, hormone-controlled, stress-induced, and plant growth and developmental regulators. Among these, we found that light-responsive elements appeared at the highest frequency in the promoter region, implying that *B. rapa TCPs* genes are modulated by light signals and operate in coordination with plant growth and development, which involves regulatory elements of MeJA-responsiveness, an important cellular regulator of abiotic and abiotic stresses during different developmental processes [54]. Hormones regulate growth and development and can also signal in response to stress (e.g., ABA) [55]. It was hypothesized that *TCPs* may affect stress tolerance in *B. rapa*. During chilling stress, transcription factors induce cold-responsive genes by attaching to promoter elements, modulating signaling cascades, and improving the cold stress of the plant [33]. *ZmTCP42* is a drought-related *TCP* gene identified from maize [28]. Most of the *TaTCP* genes identified in wheat were expressed in different parts and stages of the plant. Analyses have shown that these elements are essential in the growth of plants.

**Figure 5:**
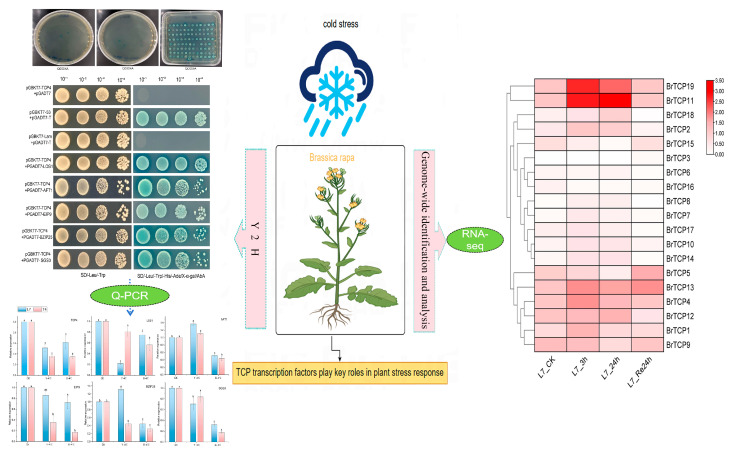
Graphical article summary. Different lowercase letters indicated significant differences among different treatments (*p* < 0.05).

Protein interactions also play an important role in protein functional differentiation. In this study, we screened TCP4 interacting proteins by constructing a yeast two-hybrid library, and the screened interacting proteins were re-screened and validated one-to-one using GO annotation and KEGG functional analysis. The results showed that the six candidate proteins, LOS1, AFT1, EIP9, BZIP25, SGS3, and TCP4, had an interaction relationship. This research offers a foundational basis for further research in the analysis of the Br*TCP* gene. The low temperature expression of the interacting proteins was analyzed, and the research showed that the expression of interacting protein genes in different tissues was different after cold stress. The mode expression of Longyou 7 in the roots was higher than that observed in Tianyou 4 after 8 h of low-temperature treatment at −4 °C, and the expression of EIP9 in the roots of Longyou 7 was about four times that of Tianyou 4. The expression of LOS1 in Tianyou 4 leaf was four times that of Longyou 7, which was found to be similar to the expression of the pattern in tomato (*Lycopersicon esculentum*). The expression patterns in different sites and the expression of individual *TCP* genes vary greatly between sites [56] (Figure 5). The expression pattern of the *CsTCP* gene in plants is also like that of the *ZmTCP* gene in corn, which contains many *TCP* genes [28]. These results indicate that *BrTCP* plays different regulatory roles in a variety of physiology, biochemistry, and stress resistance processes.

## 4. Materials and Methods

### 4.1. Materials and Treatments

Winter rapeseed (*Brassica rapa* L.) is an important overwintering oil crop that is widely planted in northwestern China. It can survive harsh winter conditions (−32 °C) and is, therefore, considered a good genetic resource for cold hardiness studies. In this study, the overwintering rate of the strong cold-resistant variety “Longyou 7” was above 80%; moreover, the overwintering rate of the weak cold-resistant variety “Tianyou 4” was between 40 and 80%. The overwintering rate of different cold-resistant varieties varies greatly. In view of this, we chose two varieties with different cold resistance rates, “Longyou 7” and “Tianyou 4”, for this study [5,57]. Uniform, healthy, and plump seeds are selected and placed on Petri dishes for sprouting. After the seeds germinated, they are planted in pots, which are incubated in a light-controlled incubator (14 h light, 25 °C; 10 h dark, 20 °C). Seedlings that grow to the five-leaf stage and grow consistently are treated with low temperature. The cold treatment is initiated by gradually reducing the temperature from 25 °C to −4 °C. Plants are induced at 10 °C and 4 °C for 48 h, respectively. Subsequently, they are treated at −4 °C for 8 h. The foliage and root systems from plants cultivated at 25 °C (serving as the control) and −4 °C (serving as the treatment) are harvested, immediately put into liquid nitrogen and frozen, and preserved at −80 °C for quantitative analysis of the utilized extracted RNA. The experiment is conducted with three biological replicates.

### 4.2. Genome-Wide Identification and Physicochemical Properties of TCP Genes

The closely related *B. rapa* genome and gene structure data were obtained from the Ensembl Plants website (https://plants.ensembl.org/index.html, accessed on 22 April 2024) [58]. The TCP transcription factor domain (PF03634) was acquired from the Pfam website (http://pfam.xfam.org/family/PF03634, accessed on 22 April 2024) [59]. Nineteen members of the *TCP* gene family were identified through Blast screening and Batch CD-Search (http://www.ncbi.nlm.nih.gov/Structure/bwrpsb/bwrpsb.cgi, accessed on 30 May 2024) [29] by TCP transcription factor members and visualized for mapping [60]. The amino acid count, molecular weight, theoretical isoelectric point (pI), instability index, aliphatic index, and grand average of hydropathicity (GRAVY) of TCP proteins were calculated using the ExPASy online tool (http://web.expasy.org/protparam/, accessed on 23 July 2024) [30]. Subcellular localization prediction of 19 protein sequences was performed using the Cell-PLoc 2.0 (http://www.csbio.sjtu.edu.cn/bioinf/Cell-PLoc-2/, accessed on 23 July 2024) website [61].

### 4.3. Analysis of TCPs Gene Family Structure, Motif, Domain, and Cis Elements

The GFF file of *B. rapa* and the nucleotide sequences of the *TCP* family of the screened *B. rapa* were analyzed using TBtools V2.110. MEME [29] was used to analyze the motif of the TCP transcription factor gene family in *B. rapa*. The protein sequences of 19 *TCPs* family members were uploaded to the online analysis software Batch CD-Search (http://www.ncbi.nlm.nih.gov/Structure/bwrpsb/bwrpsb.cgi, accessed on 28 July 2024) for domain analysis [19]. The promoter analysis of the 2 kb region upstream of the start codon of the TCP transcription factor gene in *B. rapa* was carried out with PlantCare software (PlantCARE, a database of plant promoters and their cis-acting regulatory elements, accessed on 20 August 2024), an online prediction website [62]. Finally, TBtools software was used to visualize and map the *TCPs* family gene architecture, motif, and cis-acting elements in *B. rapa* [60].

### 4.4. Phylogenetic Analysis of the TCP Proteins

Nineteen finalized *TCPs* gene family members and published TCP protein sequences (including 24 AtTCP and 22 OsTCP) were used (Supplementary Table S1) [31]. The *TCPs* gene family in *B. rapa*, *A. thaliana*, and rice were analyzed using the Neigh-Joining (NJ) method in MEGA11 [30]. The phylogenetic tree of the *TCPs* gene family was enhanced using the ITOL website (https://itol.embl.de, accessed on 26 September 2024) [63].

### 4.5. Collinearity Analysis and Mapping of TCPs Genes on Chromosomes

Members of the *TCPs* gene family were analyzed for interspecies and intraspecies collinearity using the Dual Systeny Plot and Advanced Circos tools of TBtools [60]. TBtools software was used to determine the chromosomal location information of TCP transcription factor gene family members in *B. rapa* and visualized with TBtools software.

### 4.6. Self-Activation and One-to-One Verification of BrTCP4

The positive control plasmid pGBKT 7-53 + pGADT 7-T and negative control plasmids pGBKT 7-Lam + pGADT 7T and pGBKT 7-TCP4 + pGADT 7 were engineered and introduced into the yeast strain Y2H Gold. The transfected strains were cultivated on DDO/X (SD/-Leu/-Trp/-X-a-gal) medium and incubated in an inverted orientation at 30 °C for approximately three days. Individual colonies were then selected and cultivated on QDO/X/A medium at a constant temperature of 30 °C for 3–5 days. Self-activation of pGBKT 7-TCP4 was observed based on yeast growth. Blue positive clones were picked for one-to-one verification.

### 4.7. RNA Extraction and Real-Time Fluorescence Quantitative PCR

The total RNA extraction kit of Beijing TIANGEN Company was used to extract the total RNA of Longyou 7 and Tianyou 4 of *B. rapa*. The purity was detected by 1% agarose gel electrophoresis, the concentration was measured using a nucleic acid analyzer, and the cDNA was synthesized using a reverse transcription kit (Beijing TIANGEN, Beijing, China). It was stored in the refrigerator at −80 °C for later use. The gene sequences of the interacting proteins were screened, and the primers were designed, and the primer sequences are shown in Supplementary Table S2. The upper and lower primers of the internal reference gene actin were designed with reference to previous studies [64]. The experiments were performed according to TIANGEN’s FastReal qPCR PreMix (SYBR Green) (Tiangen, Beijing, China) reagent instructions, and relative gene expression was calculated with reference to 2^−ΔΔCT^.

## 5. Conclusions

This study identified 19 *BrTCP* genes in the *B. rapa* genome, followed by genome-wide analyses. The 19 *BrTCPs* members were unevenly distributed on seven chromosomes and were divided into two clades and three clades, as follows: CIN, PCF, and CYC/TB 1 by phylogenetic analysis. We further examined the gene structures and discovered that most *AsTCP* genes lacked introns, suggesting that their genomic organization was highly conserved. *BrTCPs* contain light-responsive, hormone-responsive, stress-responsive, and plant growth and development elements, and we found that the frequency of light-responsive elements was the highest in the promoter region, indicating that the *TCPs* gene of *B. rapa* was regulated by light signals and worked synergistically with plant growth and development. Comprehensive analyses of gene structure, protein physicochemical properties, and conserved structural domains indicated that different subfamilies have different characteristics. Most *BrTCPs* gene members from the same subfamily displayed the same motifs and cis-regulatory elements at their promoters. Five *BrTCP4* interacting proteins were screened via a yeast two-hybrid experiment, and one-to-one verification was carried out. It was found that TCP4 interacted with five candidate interacting proteins in yeast cells. The expression level of interacting protein genes in different tissues was different after cold stress. Through transcriptome data, the *BrTCPs* family members of *B. rapa* had significant responses under low-temperature stress, indicating that they were important regulators in the molecular network mechanism in response to stress.

In summary, this investigation establishes a basis for subsequent studies on the role of *BrTCPs* in *B. rapa*, which is of great significance for the in-depth study of the *BrTCP4* gene to improve the resistance of *B. rapa* and lays the groundwork for the investigation of *TCP* genes.

## Figures and Tables

**Table 1:** Physicochemical properties of *BrTCPs* gene in *B. rapa.*

Gene Name	Sequence ID	Chromosomes	Number of Amino Acids	Molecular Weight/kDa	PI	Instability Index	Aliphatic Index	Hydrophilia	Subcellular Localization
*BrTCP15*	Bra004097.1	A07	347	39,379.98	5.83	49.42	64.32	−0.788	Nucleus
*BrTCP18*	Bra010789.1	A08	308	34,410.52	6.61	64.29	62.08	−0.921	Nucleus
*BrTCP13*	Bra027284.1	A05	406	44,035.42	6.99	64.44	54.16	−0.791	Nucleus
*BrTCP19*	Bra032365.1	A09	318	35,327.53	7.8	51.7	61.7	−0.929	Nucleus
*BrTCP6*	Bra029344.1	A02	301	33,796.16	6.5	47.54	64.12	−0.791	Nucleus
*BrTCP8*	Bra001032.1	A03	538	59,433.64	8.61	47.33	72.27	−0.354	Nucleus
*BrTCP10*	Bra012990.1	A03	366	40,698.22	6.18	56	59.15	−0.904	Nucleus
*BrTCP4*	Bra001579.1	A03	407	44,472.92	7.49	67.78	54.96	−0.817	Nucleus
*BrTCP11*	Bra012600.1	A03	382	42,262.93	7.12	45.39	52.15	−0.979	Nucleus
*BrTCP14*	Bra039158.1	A05	309	34,451.32	7.21	56.7	64.3	−0.706	Nucleus
*BrTCP2*	Bra021586.1	A01	350	38,203.22	6.81	75.02	56.91	−0.719	Nucleus
*BrTCP1*	Bra037579.1	A01	285	32,660.73	9.48	43.63	56.11	−0.974	Nucleus
*BrTCP7*	Bra005967.1	A03	246	27,367.45	6.49	51.36	76.54	−0.518	Nucleus
*BrTCP16*	Bra004212.1	A07	341	38,786.27	6.14	57.44	64.05	−0.874	Nucleus
*BrTCP3*	Bra034010.1	A02	346	39,308.67	5.5	48.92	65.03	−0.823	Nucleus
*BrTCP9*	Bra001710.1	A03	425	48,457.23	8.37	54.8	62.64	−0.86	Nucleus
*BrTCP17*	Bra030952.1	A08	345	37,595.34	6.74	66.6	54.12	−0.786	Nucleus
*BrTCP5*	Bra038350.1	A02	335	38,247.78	8.58	57.63	62.3	−0.881	Nucleus
*BrTCP12*	Bra018280.1	A05	349	38,727.41	6.34	50.44	51.15	−0.87	Nucleus

## Data Availability

Data from this study can be found in the article and Supplementary Materials.

## Supplementary Materials

The following supporting information can be downloaded at: https://www.mdpi.com/article/10.3390/ijms252413592/s1.
