# Supplementary material for: Genome-Wide Identification and Analysis of BrTCP Transcription Factor Family Genes Involved in Cold Stress Tolerance in Winter Rapeseed (Brassica rapa L.)
Source: Int J Mol Sci. 2024 Dec 19;25(24):13592. doi: 10.3390/ijms252413592 (PMC11678751; doi:10.3390/ijms252413592)
Supplement: Supplementary file 1 [file ijms-25-13592-s001.zip › Supplementary Table S2.pdf]

Supplementary Table S2 Real-time fluorescent quantitative PCR primers

Primer name	Sequence（5' to 3'）
q-TCP4-F	TTCCTCCACCCTCCAGCACC
q-TCP4-R	GCTTTGCTCGGCCGGT
q-LOS1-F	TGAGCTTCGTAGGATTATGGAT
q-LOS1-R	ATAAGGTACTCGTTACCATCTCT
q-AFT1-F	AGTACGTCTACATGGCGAAGCT
q-AFT1-R	TCCACCTTAGATCTGTAACCCTT
q-EIP9-F	ATCGGTGGCTCGTAACCTCAGAG
q-EIP9-R	GCCCGAGACGGAGGACGGAGTAC
q-BZIP25-F	TGTCGATGATTTGACCGACTCCT
q-BZIP25-R	GTGGAAAGAGACTGCTCGGACT
q-SGS3-F	TAAGGGAAAGAACGTCTCTTCGGGT
q-SGS3-R	GCTCCGCGACCAGTAGAGACTT
Actin-F	TGTGCCAATCTACGAGGGTTT
Actin-R	TTTCCCGCTCTGCTGTTGT
